# Bio-efficacy of new long-lasting insecticide-treated bed nets against *Anopheles funestus* and *Anopheles gambiae* from central and northern Mozambique

**DOI:** 10.1186/s12936-015-0885-y

**Published:** 2015-09-17

**Authors:** Ana Paula Abílio, Pelágio Marrune, Nilsa de Deus, Francisco Mbofana, Pedro Muianga, Ayubo Kampango

**Affiliations:** Laboratório de Entomologia, Instituto Nacional de Saúde (INS), Av. Eduardo Mondlane/Salvador Allende, no 1008, C.P. 246 Maputo, Mozambique; Programa Nacional de Controlo da Malária (PNCM), Direcção Provincial de Saúde de Cabo Delgado, Cabo Delgado Province, Mozambique; Departamento de Pesquisa, Instituto Nacional de Saúde (INS), Av. Eduardo Mondlane/Salvador Allende, no 1008, C.P. 246 Maputo, Mozambique; Direcção Nacional de Saúde Pública (DNSP), Av. Eduardo Mondlane/Salvador Allende, no 1008, C.P. 246 Maputo, Mozambique; Abt Associates Inc/AIRS, Zambezia Province, Mozambique

**Keywords:** LLINs, Bio-efficacy, *Anopheles funestus*, *A. gambiae*, Insecticide resistance, Insecticide content

## Abstract

**Background:**

Long-lasting insecticide-treated nets (LLINs) are one of the main methods used for controlling malaria transmission in Mozambique. The proliferation of several types of LLINs and the re-emergence of insecticide resistance in the local vector populations poses challenges to the local malaria control programme on selecting suitable insecticide-based vector control products. Therefore, this study evaluated the insecticide susceptibility and bio-efficacy of selected new LLINs against wild populations of *Anopheles funestus* sensu lato and *A. gambiae s.l.* from Northern and Central Mozambique. The study also investigated whether the insecticide contents on the LINNs fabrics were within the WHOPES recommended target range.

**Methods:**

The susceptibility of 2–5 day old wild female *A. funestus* and *A. gambiae* sensu stricto against the major classes of insecticides used for vector control, viz: deltamethrin (0.05 %), permethrin (0.75 %), propoxur (0.1 %), bendiocarb (0.1 %) and DDT (4 %), was determined using WHO cylinder susceptibility tests. WHO cone bioassays were conducted to determine the bio-efficacy of both pyrethroid–only LLINs (Olyset^®^, Permanet 2.0^®^, NetProtect^®^ and Interceptor^®^) and, Permanet 3.0^®^ a combination LLIN against *A. funestus s.s*, from Balama, Mocuba and Milange districts, respectively. The bio-efficacy of LLINs against the insectary-susceptible *A. arabiensis* (Durban strain) was assessed, as well. Untreated bed net swatches were used as negative controls. Chemical analyses, by high performance liquid chromatography, were undertaken to assess whether the insecticide contents on the LLINs fabrics fell within recommended target dose ranges. The frequency of *kdr* gene mutations was determined from a random sample of *A. gambiaes.s.* from both WHO susceptibility and cone bioassay experiments.

**Results:**

*Anopheles funestus* from Balama district showed resistance to deltamethrin and possible resistance to permethrin, propoxur and bendiocarb, whilst *A. gambiae* from Mocuba district was susceptible to deltamethrin, bendiocarb and propoxur. There were no *kdr* mutants found in the sample of 256 *A. gambiae* tested. Overall, 186 LLIN swatches were tested. Mosquitoes exposed to Olyset^®^ had the lowest knockdown (±standard error) and mortality rate (±standard error) in all studied sites regardless of vectors species tested. Permanet 3.0 showed the highest bio-efficacy independent of vector species tested and level of insecticide resistance detected. All types of LLINs effectively killed susceptible *A. arabiensis* Durban strain. The insecticide content of Olyset^®^ and Permanet 2.0^®^ was higher than the target dose but NetProtect^®^ had a lower insecticide content than the target dose.

**Conclusion:**

The study shows evidence of considerable heterogeneity in both insecticide susceptibility and the level of bio-efficacy of commonly available types of LLINs against wild *A. funestus* and *A. gambiae* from Balama, Mocuba and Milange districts, located in north and centre of Mozambique. The findings suggest that vector control approaches combining different types of insecticides might help to tackle the apparent problem of pyrethroid resistance in the vector populations from these three sites. Results from bioassays on laboratory-susceptible *A. arabiensis* strongly suggest that LLINs can offer some protection against susceptible malaria vectors.

**Electronic supplementary material:**

The online version of this article (doi:10.1186/s12936-015-0885-y) contains supplementary material, which is available to authorized users.

## Background

Long-lasting insecticide-treated bed nets (LLINs), often in association with indoor residual spraying (IRS), have for decades contributed to the reduction of malaria burden in sub-Saharan Africa [1, 2]. Notwithstanding recent reductions in morbidity and mortality, due to these interventions the disease remains a major problem of public health in Mozambique. The disease is responsible for nearly 45 % of the all cases observed among hospital outpatients and approximately 56 % of internments in paediatric wards [3]. Despite a decline the rate of mortality of malaria remains high, accounting for approximately 26 % of all hospital deaths [4]. LLINs continue to be the key measure for vector control in rural settings throughout the country and, since the introduction of mass distribution campaigns in 2000, it has been estimated that more than 7.6 million LLINs have been distributed, both by the Ministry of Health and partners [4]. Recently, a proliferation of several brands of LLINs in both rural and city markets has been observed. These largely derive from donations from public, private and civil organisations. Despite being beneficial to the population in needing of protection, an uncontrolled variety of nets might inadvertently, contribute to the development and spread of new foci of pyrethroid resistant strains of the local vector populations. Evaluation of LLINs against local vectors in laboratory and field studies should be performed before mass distribution of any LLIN. Moreover, studies have reported that the chemical contents of some brands of LLIN occasionally differ significantly from the recommended target doses [5]. These findings, emphasize the necessity for scrutiny and careful selection of insecticidal-based control measures since the exposure of local vectors to either sub-lethal or higher doses than that recommended for public health pesticides might potentially exacerbate the problem of insecticide resistance, as shown elsewhere [6, 7].

*Anopheles funestus*, *A. gambiae* sensu stricto (*s.s.*) and *A. arabiensis* are the most important malaria vectors found in Mozambique [8–11], whilst *A. merus* has been reported as playing secondary role on malaria transmission along the coastal regions [12].

High levels of phenotypic and metabolic resistance against the pyrethroids, deltamethrin, permethrin and alpha-cypermethrin and the carbamates propoxur and bendiocarb, have been reported in *A. funestus* from southern Mozambique [13]. However, the mosquito remained fully susceptible to DDT and malathion [14]. Resistance to lambda-cyhalothrin, permethrin and bendiocarb was reported among *A. funestus* from Zambézia Province, in the Central region [15], whereas low levels of pyrethroid and malathion resistance was detected in the provinces located in South (Maputo, Gaza and Inhambane) and Centre (Zambezia and Manica) of the country [16]. Published data on the status of insecticide susceptibility in the vector populations from Northern regions remain limited, notwithstanding, in 2006, Casimiro and colleagues [16] have reported full susceptibility to pyrethroids, carbamates and DDT in the population of *A. funestus* from Pemba city, Cabo Delgado Province; in *A. gambiae s.s.* and *A. arabiensis* from Namialo district and Nampula city, respectively, both at Nampula Province. This distribution of the patterns of malaria vector resistance against the major classes of insecticides, suggests that site-based evidence must be obtained to improve the sustainability of vector control programmes, as recommended in the WHO’s Global Plans for Insecticide Resistance Management and Vector Control [17]. Therefore, laboratory study was conducted to evaluate the response of malaria vectors from Central and Northern Mozambique to selected types of WHOPES-recommended LLINs. The current status of vector susceptibility to selected insecticides from all major classes of insecticides, currently used for vector control, was also assessed, as well as, the concentration of insecticide on LLINs fabrics. The results are discussed with respect to current malaria control policies in Mozambique.

## Methods

### Description of study sites

The study was undertaken during the dry season, from June to August 2012, in Cabo Delgado (northern region) and Zambezia provinces (central region of Mozambique). In Cabo Delgado province larvae survey were undertaken in Balama district (13°20.914′S, 38°34.183′E), located in the southern part of the province, whilst in Zambezia larvae were collected in Mocuba (16°51.00′S, 36°59.00′E) and Milange districts (16°5.810′S, 35°46.325′E) both located in the central and northeast part of Zambézia province, respectively. The three districts are among those having the highest malaria prevalence (≥40 %) in the country [18] with a low level of interventions [19]. Balama district is located at an altitude ranging from 200 to 570 m above the sea level. The climate is semi-arid with a rainy season from December to March. The mean annual precipitation ranges from 800 to 1200 mm, occasionally reaching a maximum of 1500 mm in those villages closest to the coast. The monthly air temperatures fluctuate from 20 to 25 °C. The Ruassa river is one of the most important sources of surface water in the district. The district hydrography has been dominated by underground rivers, which sometimes give rise to dispersed water bodies (locally known as Ndabo) due to either manmade excavations or through cracks that reach the surface.

Mocuba district is located at an altitude varying from 200 to 400 m above sea level. The wet season is from November to February, whilst the dry season ranges from March to October, between which some irregular rainfalls also occur. The mean annual rainfalls varies from 850 to 1300 mm and the mean monthly air temperature varies from 20 to 27 °C. Licungo and Lugela rivers are the most important sources of permanent water in the district.

Milange district is located at the northeast region of Zambézia province at an altitude varying from 200 to 1000 m above sea level. The district is boarded to the southeast by Mocuba district. The annual precipitation ranges from 800 to 1400 mm. The rainy season occurs between November and May and the mean monthly air temperature fluctuates from 24 to 26 °C.

In all three districts, during the wet season, the mean relative humidity varies from 60 to 80 %.

The people residing in the study sites are mainly subsistence farmers who grow crops such as rice, maize, beans, and manioc and cotton on the banks of small streams or rivers. Most houses are built of bamboo reinforced with mud and covered by either thatched roofs or corrugated zinc sheets. *Anopheles funestus* is the most common malaria vector in Balama and Milange district whilst *A. gambiae* sensu lato (*s.l.*) is the most common in Mocuba district. Other *Anopheles* and culicinae species, such as *A. tenebrosus*, *A. pharoensis*, *Mansonia* spp. and *Culex* spp. occur also.

### Mosquito collection

Mosquito larvae were collected in both known and potential breeding sites located along the main rivers and water collections usually found in the three districts. Larvae were collected using pipettes, dippers and bowls, depending on whether the breeding site was small or large one [20].

**Fig. 1:**
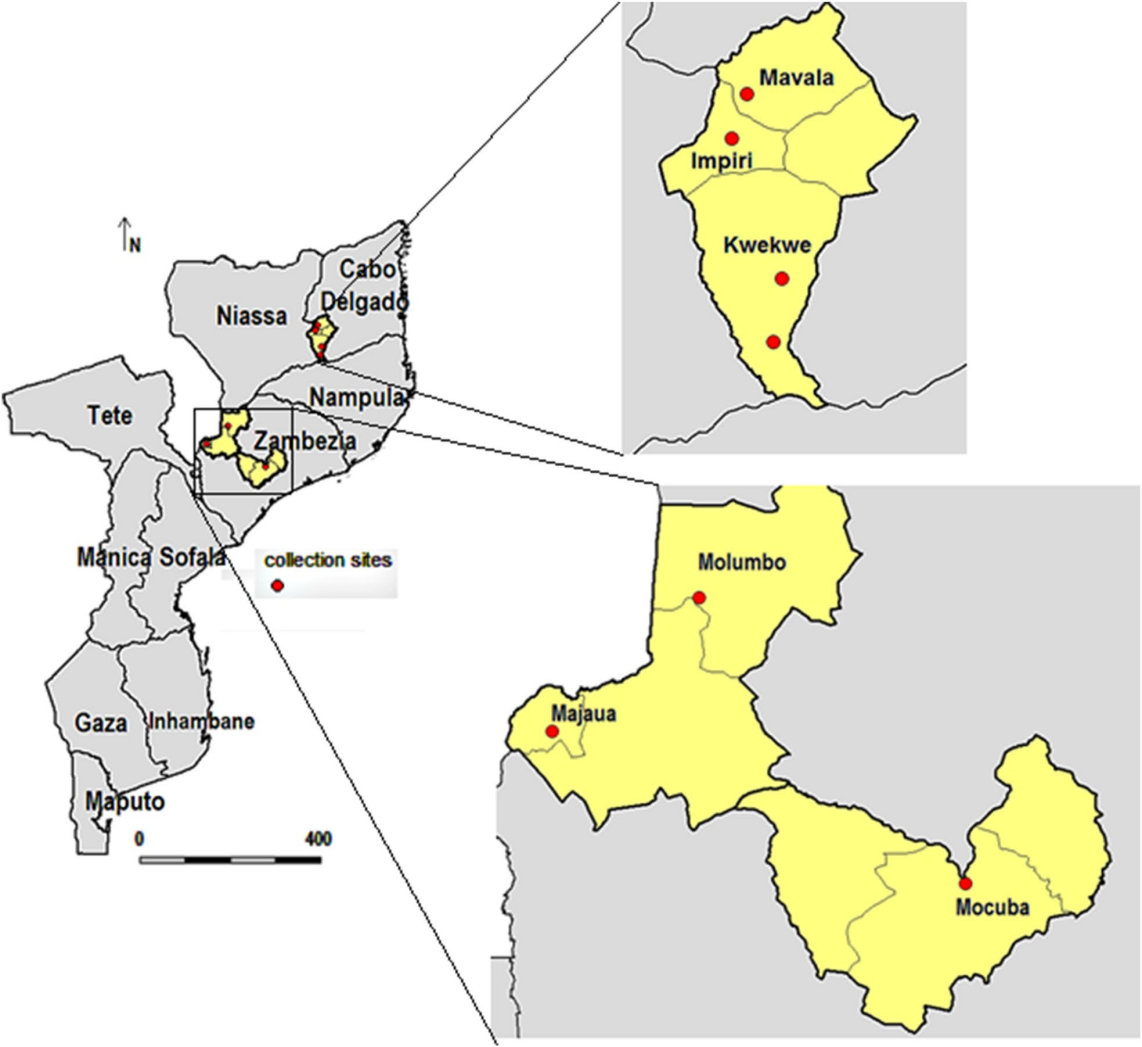
Map of Mozambique showing the geographical location of the sites where *Anopheles funestus* (Balama and Mocuba district) and *Anopheles gambiae* (Milange district) larvae were collected. In Balama district, larvae were collected in Malava, Impiri and Kwekwe villages; in Milange district larvae were collected in Majaua and Molumbo villages and in Mocuba district larvae were collected in Mocuba town

In Balama district larvae were collected in four breeding sites; two situated in Kwekwe village (breeding site 1: 13°45.567′S; 38°24.767′E and breeding site 2: 13°57.139′S; 38°23.314′E) and the other two in Mavala (13°11.776′S; 38°18.345′E) and Impiri (13°19.861′S; 38°15.684′E) villages. In Mocuba district larvae were collected in Mocuba city (16°50.997′S; 36°59.000′E), whilst in Milange district collections were carried out in Majaua (16°16.919′S; 35°26.998′E) and Molumbo (15°47.301′S; 35°59.741′E) villages (Fig. 1).

Larvae collected in Balama district were brought to the insectary located in Pemba city, the capital of Cabo Delgado province, whilst those collected in Mocuba and Milange districts were brought to the insectary located in the Quelimane city, capital of Zambezia province. Larvae were transferred into bowls and held in the insectary at room temperature and humidity of 25.1 ± 2 °C and 80 ± 5.4 %, respectively, until eclosion to adult mosquitoes. Newly emerged adult females were sorted and identified morphologically according to available taxonomic keys [21]. *Anopheles funestus* and *A. gambiae s.l.* were kept in separate cages. Morphological identification was posteriorly confirmed by PCR analysis, for members of *A. funestus* group [22] and the *A. gambiae* complex [23].

### Insecticide susceptibility tests

WHO susceptibility tests [24] were conducted to determine the susceptibility of collected vectors against permethrin (0.75 %), deltamethrin (0.05 %), bendiocarb (0.1 %), propoxur (0.1 %) and DDT (4 %). Only insecticides used to treat LLINs, as well as, those insecticides that have already been used or are currently being used for IRS. Twenty-five sugar-fed, 2–5 years old, females were transferred into testing cylinders containing papers impregnated with insecticide. The knockdown rate of mosquito exposed to the insecticides was recorded each 10 min, over 1 h exposure-period. At least four replicates were obtained for each type of insecticide tested, giving a minimum of 100 mosquitoes per insecticide. Concurrently, 50 mosquitoes (25 per cylinder) were exposed to papers impregnated with mineral oil to act as negative controls. Mosquitoes were later transferred into recovery cups and provided with cotton wool soaked in 10 % glucose solution and the final mortality was recorded 24 h later. If the mortality rate in the control cups was between 5 and 20 %, the final mortality rate was adjusted according to Abbott’s formula. When the mortality rate in the controls was >20 %, the test was discarded. Vectors were considered as being susceptible to a given insecticide if mortality rate was ≥98 %, resistant if mortality <80 % or possibly resistant if mortality was between 80 and 98 %. Mosquitoes from this test were later stored in tubes continuing silica gel and, a random sample of 194 (94 *A. funestus* and 100 *A. gambiae s.l*) mosquitoes was taken for molecular identification of vector complexes members [23]. Those specimens identified as either *A. gambiae* or *A. arabiensis* were later screened for the presence of target-site resistance *kdr* (East and West) mutations by allele-specific polymerase chain reaction, as suggested by Martin-Torre et al. [25] and Ranson et al. [26]. These mutations have often been found associated with pyrethroid/DDT cross-resistance in populations of *A. gambiae s.s.*, *A. arabiensis* and other vectors [27] but not reported in members of the *A. funestus* group [28].

### Extraction and preparation of LLINs sub-samples

The main goal was to determine the bio-efficacy and insecticide content of LLINs available in Mozambique. Five types of rectangular LLINs were investigated namely: pyrethroid-only Olyset^®^ (polyethylene fabric incorporated with 20 g/kg of permethrin), Permanet 2.0^®^ (polyester fabric coated with 55 mg/m^2^ of deltamethrin), NetProtect^®^ (polyethylene incorporated with 1.8 g/kg of deltamethrin), Interceptor^®^ (polyester coated with 200 mg/m^2^ of alpha-cypermethrin) and combination LLINs Permanet 3.0^®^. Permanet 3.0^®^ is made mainly of polyethylene fabric incorporated with 2.1 g/kg ± 25 % of deltamethrin alone (on the upper sides) and 4 g/kg ± 25 % of deltamethrin combined with 25 g/kg of a synergist piperonil butoxide (PBO) on the roof and coated with 2.8 g/kg ± 25 % of deltamethrin on the lower sides, also called borders. The lower sides are reinforced with polyester fabric [29, 30]. The PBO acts by enhanced the penetration rate of the insecticide deltamethrin through the insect cuticle inhibiting, thereby, the insects defence mechanisms, particularly the effect of enzymes P450 monooxygenases [31]. Olyset^®^ and Permanet 2.0^®^ are usually distributed as part of either mass or antenatal distribution campaigns whilst NetProtect^®^ and Interceptor^®^ are available for purchase at some local markets. Therefore, Olyset^®^ and Permanet 2.0^®^ were obtained through public and private partners of the Mozambique Ministry of Health, currently supporting the National Malaria Control Programme (NMCP) whereas, NetProtect^®^ and Interceptor^®^ were obtained by convenience and availability from the local markets. The hygiene conditions in the particular place of selling, as well as, the storage conditions of the LLINs was carefully inspected before proceeding with the purchasing of the nets. The combination LLINs PermaNet 3.0^®^ were kindly donated by Vestegaard Frandsen Ltd. All LLINs were carefully inspected to verify the physical integrity of the packet, manufacturing date and batch number.

Three samples of each LLIN were obtained. For each pyrethroid-only LLIN, (viz: Olyset^®^, Permanet 2.0^®^, NetProtect^®^ and Interceptor^®^), three 30 × 30 cm swatches from each long side and from the roof of the net were taken, making a total of 9 (3 × 3 LLINs) swatches per type of LLINs, whilst for the combination LLIN (PermaNet 3.0^®^), two swatches from the long lower sides (borders), two from the long upper sides and one from the roof were taken, giving a total of 15 (5 × 3 LLINs) samples. Individual samples were wrapped in aluminium foil and placed inside plastic labelled zip lock bags to prevent possible cross-contamination between sub-samples.

### WHO cone bioassay

WHO Cone bioassays were conducted with 2–5 day old sugar-fed females following standard WHO procedures [32]. Four cones, each containing five mosquitoes, were put in contact to 30 × 30 cm swatches taken from the sides and roof of pyrethroid-only LLINs (Olyset^®^, Permanet 2.0^®^, NetProtect^®^ and Interceptor^®^) and combination LLIN (PermaNet 3.0^®^). Mosquitoes were exposed for 3 min after which were transferred into recovery paper cups and provided with cotton wool soaked in a solution of 10 % glucose. Each swatch was tested twice, giving a total of 40 mosquitoes tested per swatch, i.e., 20 mosquitoes per 2 replicates. Mosquito knockdown rate (KD) was recorded every 30 min during a 1-h post-exposure period (KD 60) and the final mortality rate (MT) was determined 24 h post-exposure. The mortality rate was corrected using Abbott’s formula when mortality in the control was 5–20 %. Otherwise, if mortality rate in the control tube was >20 %, the bioassay round was discarded and a new test was conducted. A total of 360 mosquitoes (40 mosquitoes × 3 swatches × 3 LLINs samples) were used to test each type of pyrethroid-only LLIN whilst, 600 (40 mosquitoes × 5 swatches × 3 LLINs samples) were used to test Permanet 3.0^®^. A random sample of 477 (321 *A. funestus* and 156 *A. gambiae s.l.*) mosquitoes from this assay was used for molecular identification of vector complexes members and determination of *kdr* (West-East) resistance allele mutations, as above indicated [25]. Cone bioassays were also conducted against a susceptible colony of *A. arabiensis* (Durban strain) maintained at the entomology laboratory of the National Institute of Health (INS) in Maputo city. These tests were conducted at a room temperature and relative humidity of 25 ± 2 °C and 80 ± 5 %, respectively. The susceptibility status of the colony against the classes of insecticides commonly used for vector control has been assessed every 6 months. Sub-samples from an untreated bed-net were used concurrently as negative controls of the bioassays.

### Chemical analysis for insecticide contents

Additional samples of netting from the sides of pyrethroid-only LLINs and Permanet 3.0^®^ were collected for chemical analysis to determine if the insecticide content of the fabric was within the recommended target range. The insecticide content was determined through High Performance Liquid Chromatography (HPLC) using protocols developed by the Collaborative International Pesticides Analytical Council (CIPA) [33, 34]. Thus, deltamethrin was extracted in a mixture of iso-octane and 1,4-dioxane solution and the concentration was determined by normal-phase HPLC using dipropyl phthalate as internal standard and detection at 236 nm, whilst, alpha-cypermethrin was extracted with n-hexane and 1, 4-dioxane (95:5 v/v), shaken, sonicated and later filtered on a 0.45 mm Teflon membrane. Permethrin and piperonil-butoxide (PBO) were both extracted in the presence of hot xylene followed by drying, reconstitution and filtrations process before the final concentration was determined by HPLC. Insecticide concentration (IC) was calculated using the formula (An/As) × Cs × (Vn/ms), where An is the area of the insecticide peak in net sample, As is average area of the insecticide peak in the working standards (from a single point calibration prepared at the target concentration), Cs is average concentration of the working standards (mg/ml), Vn is volume of sample solution (100 ml) and ms is mass of net sample [35].

### Statistical data analysis

The significance of the differences between knockdown (KD 60) and mortality rates of mosquitoes exposed to different types of LLIN were analysed by Generalized Linear Mixed Models (GLIMM) using binomial error distribution and logit link function [36]. Initially, GLIMM tests were applied using lme4 v. 1.1–7 package [37], the type of LLIN was considered as fixed factor, whilst the sides and roof of it was considered as a random factor nested within each bed net type, so as to account for any possible non-constant variability of knockdown and mortality rates between the side of LLINs and any possible correlations between repeated measures taken from the same swatch. Subsequently, the fitted models for each study site and species tested were used to determine the significance of difference of KD 60 and mortality rate between the types of LLINs using the package multcomp v. 1.3–7 [38]. The Tukey HSD test was applied to assess the significance of the differences. The *p*-values estimated by the Tukey HSD test was adjusted to account for multiplicity and correlation between statistics using the Westfall truncated closed test procedure, implemented also with multcomp v. 1.3–7 [39]. Probit regression analysis was applied to mortality rates from the susceptibility tests to estimate the median exposure time necessary to kill 50 % (KDT_50_) and 95 % (KDT_95_) of the vector populations when exposed to each class of insecticides tested, using the package drc [38]. All statistical analysis were performed using R v. 3.1.2 [40].

### Ethical considerations

The study received ethical approval by the National Committee of Bioethics of the Mozambique Ministry of Health, under the registration number 06/CNBS/12.

## Results

### Vector populations

1680 *A. funestus* from Balama and 1670 Mocuba districts and 1720 *A. gambiae s.l.* from Milange district were used to perform cone bioassays. 10 data points of Permanet 3.0 from Milange district were missing. Additionally, 500 *A. funestus* and 400 *A. gambiae* were used to undertake the susceptibility tests against Propoxur, Deltamethrin, Permethrin, Bendiocarb and DDT.

All 415 members of the *A. funestus* group analysed by PCR were *A. funestus s.s* and all 256 *A. gambiae s.l*. were *A. gambiae s.s*, S form. Therefore we presume that these were the only two vector species in the study.

### Insecticide susceptibility

**Fig. 2:**
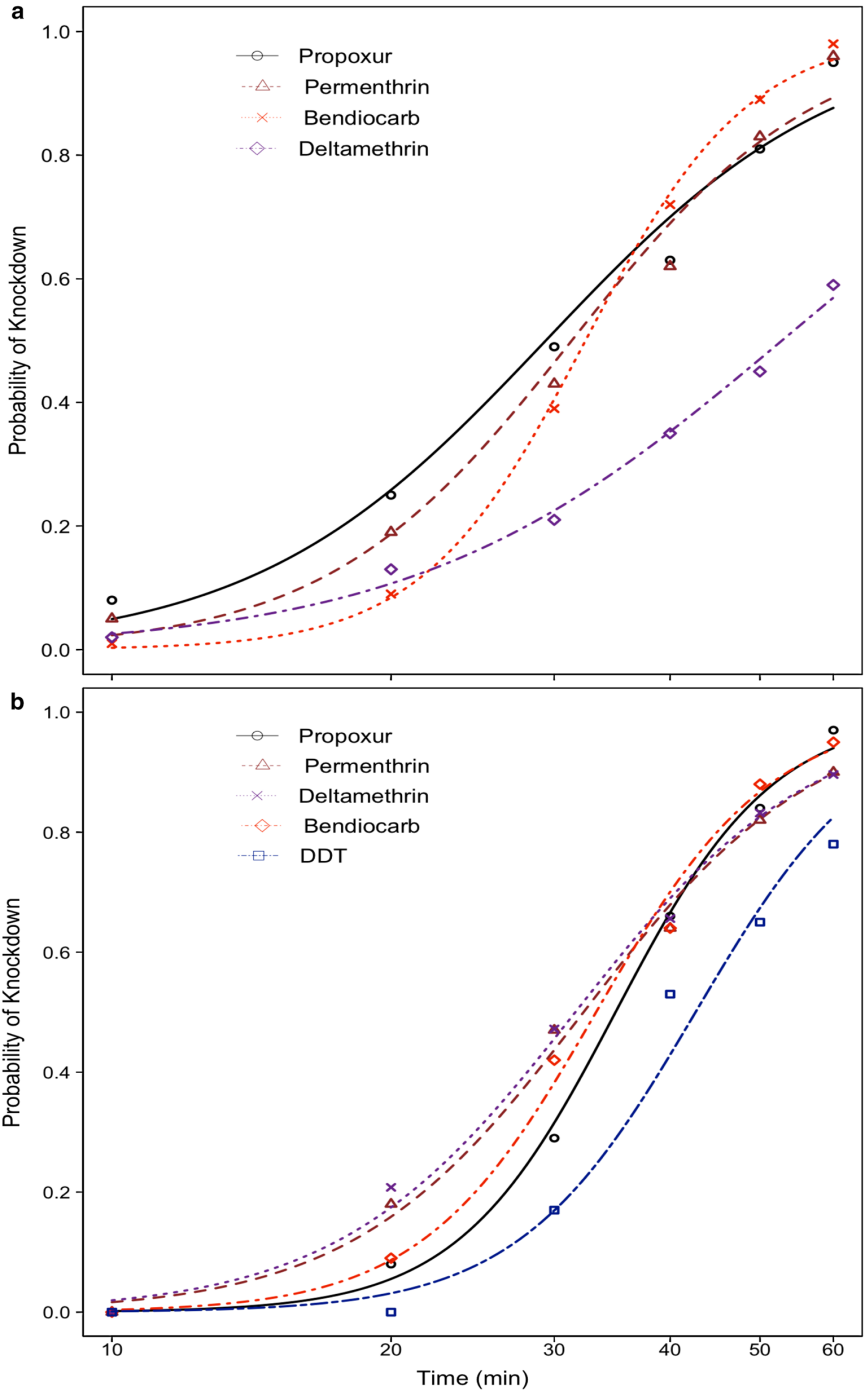
Response curves showing the probability of knockdown of *Anopheles funestus* **a** from Balama distric and *Anopheles gambiae s.s* **b** from Mocuba district exposed to selected types of insecticides over 60 min exposure-time

The knockdown rates of *A. funestus s.s.* and *A. gambiae s.s.* (henceforth *A. funestus* and *A. gambiae*) exposed to five selected insecticides are showed in Fig. 2a, b, respectively. The probability of an insect being knocked down during the first 30 min of exposure varied from 0 to 46 % (in *A. funestus*) and 0 to 50 % (in *A. gambiae*), suggesting that a high frequency of resistant strains in the two vector populations. These results were later corroborated by the estimates of the median time (in minutes) required to kill 50 % [KDT_50_ (±95 % CI)] and 95 % [KDT_95_ (±95 % CI)] of the vectors populations when exposed to the same insecticides (Tables 1, 2). There was no expressive difference between these estimates for either species. The smallest KDT_50_ estimate for *A. funestus* was observed when mosquitoes were exposed to propoxur [29.37 (27.17–31.58)], whilst the smallest KDT_95_ was observed against bendiocarb [58.84 (53.18–64.50)]. The shortest KDT_50_ and KDT_95_, in *A. gambiae* was observed with deltamethrin [31.61 (29.81–33.41)], bendiocarb [33.28 (31.55–35.01)] and propuxor [34.9 (33.24–36.61)] whilst, the shortest KDT_95_ estimate was 62.29 (56.32–68.26) obtained against bendiocarb and propoxur, respectively.

**Table 1:** Mortality rate of *Anopheles funestus* from Balama district exposed to four types of insecticides and, the estimated median time (in minutes) required to kill 50 % (KDT_50_ ± 95 % CI) and 95 % (KDT_95_ ± 95 % CI) of the vector population when exposed to the same insecticides

Insecticide	Mosquito tested	KDT_50_ (± 95 % CI)	KDT_95_ (± 95 % CI)	Mortality rate
Deltamethrin (0.05 %)	100	52.81 (47.11–58.51)	203.07 (127.33–278.81)	85
Permethrin (0.75 %)	100	31.33 (29.28–33.38)	77.08 (65.88–88.29)	97
Bendiocarb (0.1 %)	100	32.42 (30.78–34.06)	58.84 (53.18–64.50)	92
Propoxur (0.1 %)	100	29.37 (27.17–31.58)	85.92 (70.97–100.88)	94

**Table 2:** Mortality rate of *Anopheles gambiae* from Mocuba district exposed to five types of insecticides and, the estimated median time (in minutes) required to kill 50 % (KDT_50_ ± 95 % CI) and 95 % (KDT_95_ ± 95 % CI) of the vector population when exposed to the same insecticides

Insecticide	Mosquito tested	KDT_50_ (95 % CI)	KDT_95_ (95 % CI)	Mortality rate
Deltamethrin (0.05 %)	125	31.61 (29.81–33.41)	75.24 (65.82–84.65)	99.2
Permethrin (0.75 %)	100	32.26 (30.25–34.27)	75.11 (64.80–85.42)	97
Bendiocarb (0.1 %)	100	33.28 (31.55–35.01)	62.29 (56.32–68.26)	99
Propoxur (0.1 %)	100	34.9 (33.24–36.61)	62.29 (56.33–68.26)	98
DDT (4 %)	100	42.6 (40.51–44.69)	81.59 (71.40–91.77)	97

In Balama district, the mortality rates of *A. funestus* recorded 24 h post-exposure, suggest that it might be resistance to virtually all four types of insecticides tested (Table 1). In Mocuba, on the other hand, results suggested that *A. gambiae* might be resistant to permethrin, propoxur and DDT and, susceptible to deltamethrin and bendiocarb (Table 2). However, molecular analysis failed to reveal the presence of kdr gene mutant alleles in random sample of 250 specimens of *A. gambiae* tested. Susceptibility tests were not performed on mosquitoes from Milange district due to the low number of mosquitoes collected.

### Bio-efficacy of LLINs against wild-caught vector populations

**Fig. 3:**
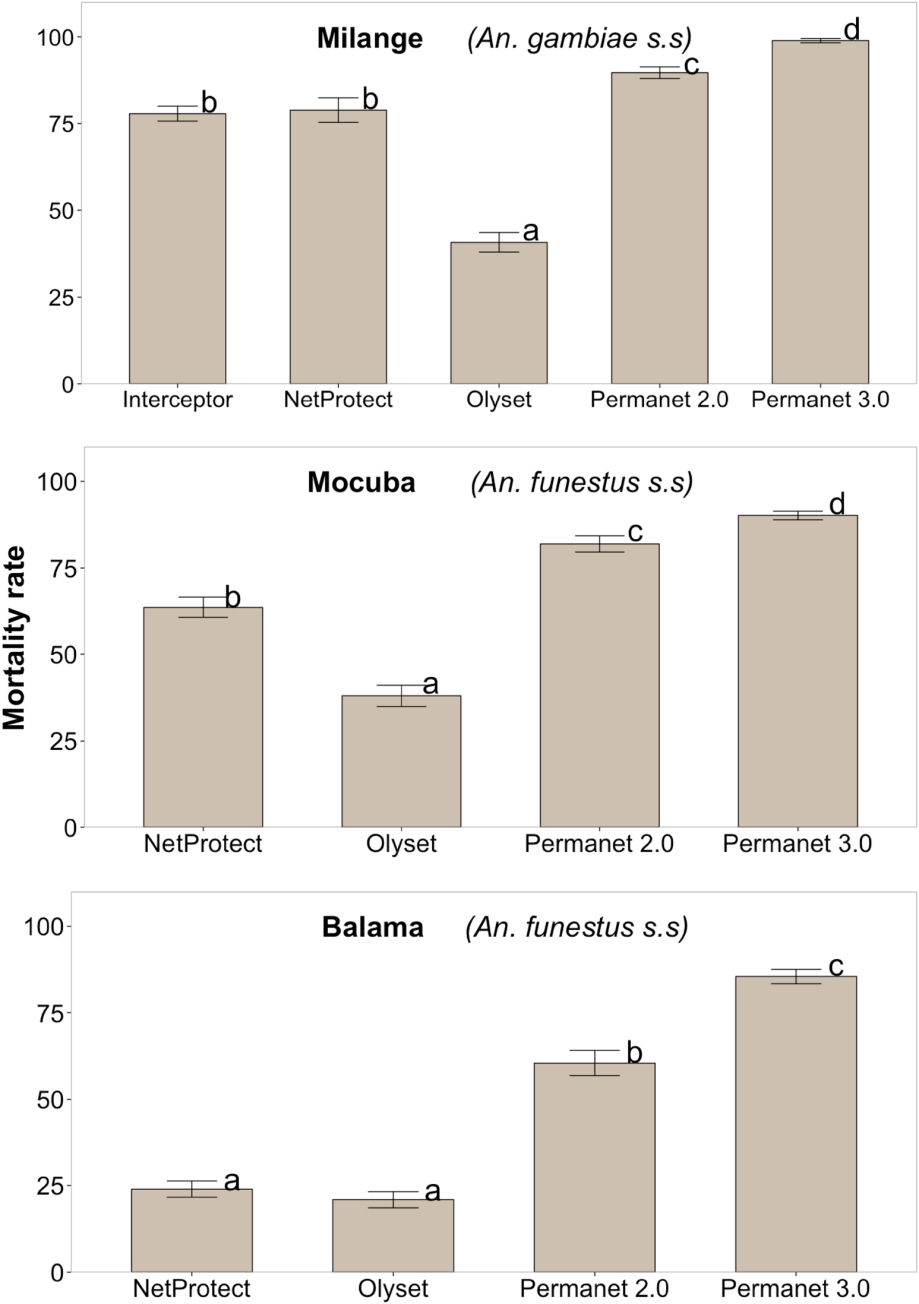
Comparison of mortality rates of *Anopheles gambiae s.s* (Milange distric) and *Anopheles funestus* (Mocuba and Balama district) mosquito females exposed to different brands of LLINs. *Letters above each bar* display the significance of the difference of Mortality rates between pairs of bed nets, obtained by TukeyHSD at 5 % significance level. Mortality rates followed by the *same letter* are not statistically significant. The *letters* were sorted starting from lower (*a*) to higher (*d*) significant Mortality rate. P-values were adjusted using Westfall procedure (see Additional file 1 for further details)

135 LLINs swatches were obtained, 90 from pyrethroid-only LLINs and 45 from combination Permanet 3.0^®^. 84 swatches were tested against *A. funestus* from Balama (42/135) and Mocuba (42/135) districts and 51/135 against *A. gambiae* from Milange district, respectively the knockdown (KD 60) and mortality rates of the two species exposed to the five types of LLINs are depicted on Table 3 and Fig. 3. There was a significant difference in both knockdown (F = 151.52, *P* < 0.0001) and mortality rates (F = 181.74, *P* < 0.0001) of mosquitoes exposed to LLINs. In addition, there was also a significant correlation between the knockdown rate and mortality rate (R^2^ = 0.857, *P* < 0.0001), when the data were stratified by species and study sites, indicating that previous exposure of mosquitoes to insecticides on bed nets explained 85.7 % of the total variation of mortality rates recorded 24 h post-exposure. Therefore, further statistical analyses were mainly focused on mortality rates as an indicator of bio-efficacy.

**Table 3:** Knockdown (KD 60 ± standard error) and mortality (±standard error**)** rates of *A. funestus* (Balama and Mocuba district) and *A. gambiae* from Milange district tested against five brands of Long-lasting insecticide-treated bed nets (LLINs)

LLINs	Mosquito tested per site	Bio-efficacy indexes	Study districts		
			Balama (*A. funestus*)	Mocuba (*A. funestus*)	Milange (*A. gambiae*)
Olyset	360	KD 60 (±se)	*35.55 (±3.15)*	*49.14 (±2.47)*	*57.5 (±2.71)*
		Mortality rate (±se)	*20.9 (±2.34)*	*38.07 (±3.07)*	*40.77 (±2.82)*
Permanet 2.0	360	KD 60 (±se)	69.72 (±3.40)	78.61 (±2.18)	91.25 (±1.30)
		Mortality rate (±se)	60.48 (±3.64)	81.94 (±2.32)	89.65 (±1.65)
Permanet 3.0	600	KD 60 (±se)	93.33 (±1.12)	85.16 (±1.43)	99.64 (±0.36)
		Mortality rate (±se)	85.5 (±2.09)	90.16 (±1.27)	98.92 (±0.61)
NetProtect	360	KD 60 (±se)	*61.38 (±2.79)*	62.22 (±2.52)	83.88 (±1.61)
		Mortality rate (±se)	*23.95 (±2.34)*	63.61 (±2.95)	78.87 (±3.56)
Interceptor	360	KD 60 (±se)	–	–	80.83 (±1.87)
		Mortality rate (±se)	–	–	77.84 (±2.16)

(–) Not tested

Highlighted cell indicates where significant difference between knockdown and mortality rate was found at 5 % significance level

In general, the pyrethroid-only Olyset^®^ (permethrin incorporated) showed the lowest bio-efficacy (mean ± standard error) against *A. funestus* from Balama (20.9 ± 2.34) and Mocuba (38.07 ± 3.07) and *A. gambiae* from Milange (40.77 ± 2.82) when compared to the same vectors exposed to other types of LLINs (Table 3). The highest bio-efficacy was observed with deltamethrin coated (Permanet 2.0^®^), deltamethrin incorporated (NetProtect^®^) and deltametrin incorporated/coated plus piperonyl-butoxide (PBO) incorporated Permanet 3.0^®^ (Table 3). The LLIN Interceptor^®^ (alpha-cypermetrin coated) was only tested against *A. gambiae* from Milange district. The mortality rate (±standard error) of *A. gambiae* exposed to Interceptor^®^ was 77.84 ± 2.16. This was similar to that obtained with Netprotect^®^ (78.87 ± 3.56; *P* = 0.839). The mortality of *A. funestus* from Balama district exposed to Netprotect^®^ (23.95 ± 2.34) and Olyset^®^ (20.9 ± 2.34; *P* = 0.129) did not differ significantly (see Fig. 3; Additional file 1, for further details).

**Fig. 4:**
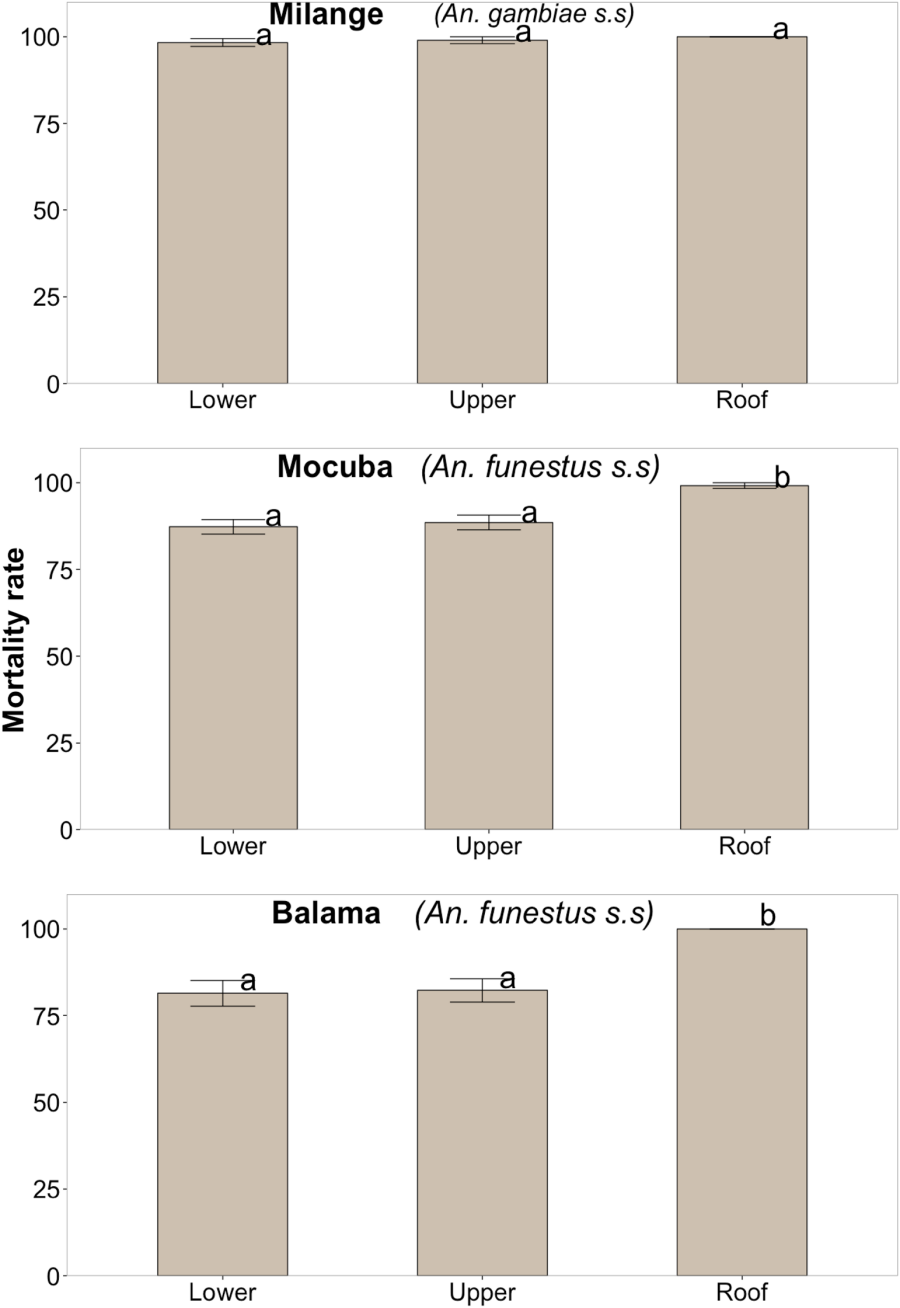
Comparison of mortality rates of *Anopheles gambiae s.s* (Milange distric) and *Anopheles. funestus* (Mocuba and Balama district) mosquito females exposed to sides and roof of Permanet 3.0. *Letters above each bar* display the significance of the difference of Mortality rates between pairs of bed nets, obtained by TukeyHSD at 5 % significance level. Mortality rates followed by the *same letter* are not statistically significant. The *letters* were sorted starting from lower (*a*) to higher (*d*) significant Mortality rate. P-values were adjusted using Westfall procedure (see Additional file 2)

Results of mortality rates of mosquitoes exposed to Permanet 3.0^®^ were stratified by site of the bed-net, namely, lower side (border), upper side and roof (Fig. 4). The mortality rate of *A. funestus* from Balama district exposed to roof sub-samples was significantly higher than of those exposed to samples from lower sides [Estimated difference ± standard error (se) = 18.54 ± 5.47; *P* = 0.003] and upper sides [Estimated difference ± se = 17.71 ± 5.46; *P* = 0.003]. A similar result was obtained with *A. funestus* from Mocuba district, i.e. estimated difference (±se) roof vs. lower side (11.88 ± 3.31; *P* = 0.0013); roof vs. upper side (10.63 ± 3.31; *P* = 0.0017). There was no significant difference of mortality rates of *A. gambiae* from Milange district, exposed to swatches from either sides of Permanet 3.0^®^ (Fig. 4; Table 4; Additional file 2).

**Table 4:** Knockdown (±standard error) and mortality (±standard error) rates of *A. funestus* (Balama and Mocuba district) and *A. gambiae s.* from Milange district exposed to different sides of combination LLIN Permanet 3.0

Permanet 3.0 sides	Mosquito tested per site	Bio-efficacy indexes	Study sites		
			Balama (*A. funestus*)	Mocuba (*A. funestus*)	Milange (*A. gambiae*)
Lower side (border)	240	KD 60 (±se)	*90 (±2.06)*	84.58 (±1.91)	100 (±0.00)
		Mortality rate (±se)	*81.46 (±3.69)*	87.29 (±2.10)	98.33 (±1.15)
Upper side	240	KD 60 (±se)	*93.33 (±1.92)*	80 (±2.60)	99 (±1)
		Mortality rate (±se)	*82.29 (±3.35)*	88.54 (±2.13)	99 (±1)
Roof	120	KD 60 (±se)	100 (±0.00)	96.67 (±1.55)	100 (±0.00)
		Mortality rate (±se)	100 (±0.00)	99.17 (±0.83)	100 (±0.00)

Highlighted cell indicates where significant difference between knockdown and mortality rate of vector was found at 5 % significance level

### Bio-efficacy against colony susceptible vectors

A total of 51 swatches (36 from pyrethroid-only LLINs and 15 from combination Permanet 3.0^®^) were tested against the colony of susceptible *A. arabiensis* (Durban strain). The knockdown and mortality rates from this bioassay indicate that all type of LLINs performed well against this mosquito (Table 5). The mortality rate (±standard error) varied from 90.36 ± 1.34 % to 100 ± 0.00 % when mosquitoes were exposed to Olyset^®^, Permanet 2.0^®^ and Permanet 3.0^®^, respectively. Comparisons between the mortality rates of wild-caught *A. funestus* and *A. gambiae* (see Table 4) and the *A. arabiensis* colony (Table 5) indicated that the mortality rate of the *A. arabiensis* exposed to both Olyset^®^ and NetProtect^®^ was two to four times higher than the rates obtained with *A. funestus* from Balama and Mocuba district and *A. gambiae* from Milange district (see Additional file 3). There was no expressive difference of the ratios between the mortality rates of susceptible and wild-caught mosquitoes exposed to Permanet 2.0^®^, Permanet 3.0^®^ and Interceptor^®^ (Additional file 3).

**Table 5:** Knockdown (±standard error) and mortality rates (±standard error) of insectary-susceptible *A. arabiensis* (Durban strain) exposed to LLINs

Type of LLIN	Mosquito tested	KD 60 ± se	Mortality rate ± se
Olyset	360	68.33 ± 2.26	90.36 ± 1.35
Permanet 2.0	360	94.72 ± 1.12	100 ± 0.00
Permanet 3.0	600	98.17 ± 0.58	100 ± 0.00
NetProtect	360	83.89 ± 1.80	99.44 ± 0.39
Interceptor	360	80.56 ± 1.63	98.84 ± 0.57

### Insecticide contents on the LLINs

Fifty-one swatches were assessed for insecticide concentration, 36 from pyrethroid-only LLINs and 15 from combination Permanet 3.0^®^. The results indicated that, the insecticide concentration on the swatches from sides (1.0 g/kg) and roof (1.0 g/kg) of NetProtect^®^ were below the target dose range (1.8 g/kg) whereas, the sides (23.2 g/kg) and roof (23.6 g/kg) of Olyset^®^ and roof (73.2 mg/m^2^) of Permanet 2.0^®^ had insecticide content above those specified by manufacturers (Table 6).

**Table 6:** Comparisons between measured and target dose of insecticide contents on swatches from sides and roof of LLINs

Net type	Active ingredient	Net section	Target mean dose	Target dose range	Measured mean dose	Measured dose within product target range?
Interceptor (IT)	Alpha-cypermethrin	Roof	200 mg/m^2^	150.0–250.0	204.2 mg/m^2^	Yes
	Alpha-cypermethrin	Sides	200 mg/m^2^	150.0–250.0	204.2 mg/m^2^	Yes
NetProtect (NP)	Deltamethrin	Roof	1.8 g/kg	1.35–2.25	1.0 g/kg	Under
	Deltamethrin	Sides	1.8 g/kg	1.35–2.25	1.0 g/kg	Under
Olyset (OL)	Permethrin	Roof	20 g/kg	17.0–23.0	23.2 g/kg	Over
	Permethrin	Sides	20 g/kg	17.0–23.0	23.6 g/kg	Over
Permanet 2.0 (PN2)	Deltamethrin	Roof	55 mg/m^2^	41.25–68.75	73.2 mg/m^2^	Over
	Deltamethrin	Sides	55 mg/m^2^	41.25–68.75	65.8 mg/m^2^	Yes
Permanet 3.0 (PN3)	Deltamethrin	Roof	4 g/kg	3.0–5.0	3.4 g/kg	Yes
	Deltamethrin	Lower side (border)	2.8 g/kg	2.1–3.5	3.0 g/kg	Yes
	Deltamethrin	Upper side	2.8 g/kg	2.1–3.5	3.1 g/kg	Yes
	PBO	Roof	25 g/kg	18.75–31.25	28.8 g/kg	Yes

## Discussion

### Vector susceptibility to insecticides

The results from WHO susceptibility tests indicate that the *A. funestus* population from Balama district, Cabo Delgado Province, has possibly became resistant to all the four types of insecticides tested two of which were pyrethroids and two carbamates viz: deltamethrin (0.05 %), permethrin (0.75 %), bendiocarb (0.1 %) and propoxur (0.1 %), respectively. Resistance against the two pyrethroids may be due the over expression of the enzymes P450 mono-oxigenases [41], whilst resistance to carbamates may be to an elevated production of acetylcholinesterase [27]. Prior to undertaking this study, there was no previous report about the susceptibility status of malaria vectors from Balama district. However, results from this study contrast with that obtained in 2006 by Casimiro et al. [16] in Pemba city, located at approximately 250 km from Balama district, which reported full susceptibility (100 % of mortality) of *A. funestus* to lambdacyalothrin (0.05 %), deltamethrin (0.05 %), propoxur (0.1 %), malathion (5 %) and DDT (4 %). The authors also detected an elevated expression of glutathione-S-transferase (GST) in the wild population of *A. funestus* compared to laboratory-resistant *Aedes aegypti* strains. As such, the resistance to DDT found in Balama district may probably be related to elevated expression of GST associated with resistance to DDT in several insect populations, including malaria vectors [27]. Recently, it has been demonstrated that a single mutation (GSTe2) in the sequence of the gene that encodes for GST in *A. funestus* from Benin, can confer resistance to both DDT and pyrethroids [42]. Previous studies from Southern Mozambique have also reported a high level of pyrethroid resistance in *A. funestus* [13] consistently associated with a high expression of cytochrome P450 mono-oxygenases [43, 44]. Unfortunately, in Mocuba city, the number of *A. funestus* collected was not enough to perform susceptibility tests, other than those used for the cone bioassay. Meanwhile, approximately 500 larvae of *A. gambiae* were collect. Adults derived from these larvae were used to perform the susceptibility tests (Fig. 2b; Table 2). Results indicate that *A. gambiae* from Mocuba city remains susceptible to bendiocarb (0.1 %), propoxur (0.1 %) and deltamethrin (0.05 %) but is possibly resistant to permethrin (0.75 %) and DDT (4 %). These findings contrast with those from Abilio and colleagues [15], who, in 2011, reported full susceptibility of *A. gambiae s.l.* to pyrethroids and DDT.

There were no *kdr* gene resistant mutants detected in a random sample of n = 256 *A. gambiae* tested, despite the susceptibility tests suggesting resistance to DDT and pyrethroids. The *kdr* resistance mechanism has been consistently associated with cross-resistance to pyrethroid and DDT in populations of *A. gambiae* and *A. arabiensis* [27]. The mechanism is yet to be identified in *A. funestus* [28]. Unfortunately, metabolic resistance assays were not carried out in this study. Therefore the insecticide resistance mechanism involved in conferring resistance among these insects is not known yet. Riveron et al. [42] have recently reported that a single amino acid change in the binding pocket of the glutathione-s-transferase epsilon 2 (GSTe2) gene confers a high level of DDT resistance and also cross-resistance to pyrethroids in *A. funestus*. The expression of GSTe2 mutation has also been widely documented in *A. gambiae* [45].

### Bio-efficacy of pyrethroid-only LLINs

This study is the first to determine the response of wild-caught malaria vectors from Central (Mocuba and Milange districts) and Northern (Balama district) regions of Mozambique to commonly available types of LLINs. The results of cone bioassay indicated that the bio-efficacy of pyrethroid-only LLINs varied significantly depending on the vectors species tested (Fig. 3; Additional file 1). Overall, Olyset^®^ and NetProtect^®^ showed a dramatically lower bio-efficacy, regardless of vector species was tested (Table 3). Permanent 2.0^®^ showed a higher bio-efficacy against both *A. funestus* from Balama and Mocuba city and against *A. gambiae* from Milange district, compared to either Olyset^®^ or NetProtect^®^. However, in Balama district Permanet 2.0^®^ had a lower bio-efficacy compared to that observed in Mocuba and Milange districts (Table 3). The lower performance of these two type of pyrethroid-only LLINs, particularly against *A. funestus* from Balama district, may be due to the existence of resistant individuals in the local vector population as demonstrated in the WHO susceptibility tests (Fig. 2a; Tables 1, 2). Olyset^®^ and Permanet 2.0^®^ have been the two main brands of LLINs usually distributed as part of mass and antenatal distribution campaigns in Mozambique. Thus, results from Balama district strongly suggest that Olyset^®^ and Permanet 2.0^®^ may not be effectively killing *A. funestus* in those regions where there are resistant population foci. Studies should be extended to other locations of Balama district in order to get the current picture on both phenotypic and metabolic insecticide resistance profile in the malaria vectors population and, thereby, be able to accurately predict the impact any control approach may have on the vector populations at district level. However, several studies have shown that LLINs still protect people against infectious mosquito bites despite insecticide resistance detected in the vector population, since the pyrethroids are also, to certain degree, repellent to mosquitoes [46] and, as long as the integrity of the fabric remains intact, the LLIN is also a physical barrier between sleepers and mosquitoes, [47]. In addition, more than 90 % of susceptible *A. arabiensis* were killed when exposed to LLINs in bioassays (Table 5; Additional file 3), suggesting that the LLINs can control susceptible mosquitoes. Interestingly, the mortality rate of *A. gambiae* from Milange exposed to both Interceptor^®^ and NetProtect^®^ was statistically similar (*P* = 0.839) (see Table 3; Fig. 3; Additional file 1); this suggest that the two types of LLINs might probably perform equally well in the field. Since they have been treated with different insecticide formulations then having both nets in use may reduce the selective pressures that favour the occurrence of resistant strains in the vector compared to the situation when a single type of insecticide or LLINs is used. Unfortunately, the bio-efficacy of Interceptor^®^ against vectors from Balama and Mocuba was not assessed. However, the knockdown and mortality rate of *A. funestus* from Furvela village, in southern Mozambique, exposed to Interceptor^®^ swatches, suggested that the vector population was resistant to the insecticide (JD Charlwood et al., *unpublished report*).

### Bio-efficacy of combination Permanet 3.0^®^

Permanet 3.0^®^ performed well against the two malaria vectors populations, irrespective of the level of resistance to pyrethroids. *Anopheles funestus* from Balama and Mocuba district exposed to swatches from the roof had the highest mortality compared to mosquitoes exposed to the upper and lower sides of the net whilst the mortality rates of *A. gambiae* from Milange district was independent of the location tested (Table 4; Fig. 4; Additional file 2). The higher mortality rates observed when mosquitoes were exposed to roofing swatches of Permanet 3.0^®^ was probably due to the presence of the synergist PBO and the higher concentration of insecticide on the fabric of the roof of the net. In southern Mozambique, Brooke and colleagues [13] managed to revert the resistance of *A. funestus* against the lambda-cyalothrin after pre-exposing the insect to PBO. This prompted the authors to suspect that the mean metabolic resistance involved at the time (in 2001) was the over expression of enzyme mono-oxygenases; later reported in *A. funestus* from Belulane district [43] and recently in *A. funestus* from Chókwè villages [44]. The higher concentration of deltamethrin in the roofing fabrics compared to sides of Permanet 3.0^®^ may have also caused higher mortality rate of mosquitoes exposed to it. However, increased insecticides concentration may be, per se, a counterproductive measure, since it can also contribute to rapid selection of resistant strains in the population, as discussed in [48]. Previous and recent field and laboratory works have reported better performance of combinations of “two-in-one” approaches, i.e. the combination of pyrethroid and non-pyrethroid insecticides applied to different parts of the bed nets [49]. However, recent reports have demonstrated that the better performance of Permanet 3.0^®^ has been only achieved with unwashed bed nets [50, 51]. These studies have also noted that the biological activity of both deltamethrin and PBO tend to reduce significantly after a few washes, despite a high concentration of the two insecticidal compounds [52], suggesting that further investigation on insecticide retention by Permanet 3.0^®^ fabrics must be done to improve the field performance of the net.

### Insecticide concentration on bed nets

Chemical analysis of swatches from the sides and roof of the nets indicated that the insecticide content from the sides and roof of Olyset^®^ and the roof of Permanet 2.0^®^ was above the target dose. On the other hand, the insecticide concentration of NetProtect^®^ was below that recommended dosage (Table 6). Intriguingly, Olyset^®^ showed a low performed against both vectors species despite high level of insecticide found. This implies that different types of insecticide resistance mechanisms are involved. Laboratory and field evidence has shown that the insecticide concentration on the fabric of a LLIN decays over time, for instance after 6 months of intensive usage and washes, as recently reported in Permanet 3.0^®^ [51] or due to bad storage. However, in the present study new nets were tested. The integrity of the packets and the expiration date of each type of LLIN were carefully verified before the extraction of the sub-samples. Therefore, the low insecticide content observed in NetProtect^®^ swatches was caused by unidentified factors. Similar studies have reported significant differences of insecticide contents between the sides and roof of Permanet 2.0^®^ [5] and Permanet 3.0^®^ [53]. These findings have obvious operational implications since the concurrent exposure of vectors to varying doses of the same insecticides might potentiate resistance in the vector [6].

All types of LLINs tested in this study performed remarkably very well against the colony of susceptible *A. arabiensis*, maintained at the insectary of the National Institute of Health (INS) in Maputo.

## Conclusion

Considerable heterogeneity in both, insecticide susceptibility and the level of bio-efficacy of commonly available types of LLIN’s was observed among pyrethroid resistant populations of wild-caught *A. funestus* and *A. gambiae* from northern and central Mozambique. The findings suggest that vector control approaches by combining different types of pyrethroid-based methods, particularly LLINs, might help to tackle the apparent problem of pyrethroid resistance in the malaria vectors such as these, as it would both increase the killing efficacy against the vectors and concurrently reduce the selective pressures favouring the occurrence of resistant strains. The on-going management of insecticide resistance in vector control programmes is, obviously, mandatory for an effective malaria control. Results from bioassays against susceptible *A. arabiensis* strongly suggested the LLINs tested will still kill susceptible mosquitoes and so can help reduce transmission. Similar studies should be extended throughout the country in order to fill the gaps in the current knowledge concerning the status of phenotypic and metabolic resistance of malaria vectors populations, as well as, to determine the extent to which vectors might respond to insecticide-based vector control approaches prior to their implementation.

## Additional files

**Additional file 1:** Results of pair-wise comparisons, obtained by TukeyHSD test, of overall mortality rates of mosquitoes exposed to different types of LLINs.
**Additional file 2:** Results of pair-wise comparisons, obtained by TukeyHSD, of mortality rates of mosquitoes exposed to different sides of Permanet 3.0.
**Additional file 3:** Ratio between the mortality rate of insectary-susceptible *Anopheles arabiensis* (Durban strain) and the mortality rate of wild-caught *Anopheles funestus* (from Balama, Mocuba district) and *Anopheles gambiae s.s.* from Milange district. The figure shows that the LLINs can still remarkably killing higher number (mortality rate > 90 %) of susceptible mosquitoes.
