# Supplementary material for: Bio-efficacy of new long-lasting insecticide-treated bed nets against Anopheles funestus and Anopheles gambiae from central and northern Mozambique
Source: Malar J. 2015 Sep 17;14:352. doi: 10.1186/s12936-015-0885-y (PMC4574012; doi:10.1186/s12936-015-0885-y)
Supplement: Supplementary file 1 — Additional file 1: Results of pair-wise comparisons, obtained by TukeyHSD test, of overall mortality rates of mosquitoes exposed to different types of LLINs. [file 12936_2015_885_MOESM1_ESM.docx]

Table 1. Pair-wise comparisons, obtained by TukeyHSD test, between the mortality rates of *A. gambiae* from Milange district exposed to different types of bed nets. P-values adjusted using Westfall procedure implemented with multcomp v. 1.3-7 package.

| **Pair-wise comparisons** | **Estimate** | **Std. Error** | **z value** | ***P values*** |
| --- | --- | --- | --- | --- |
| NetProtect vs. Interceptor | 0.04933 | 0.24337 | 0.203 | **0.83936** |
| Olyset vs. Interceptor | -1.60538 | 0.16755 | -9.582 | < 0.001 |
| Permanet 2.0 vs. Interceptor | 0.92832 | 0.21406 | 4.337 | < 0.001 |
| Permanet 3.0 vs. Interceptor | 3.2565 | 0.58111 | 5.604 | < 0.001 |
| Olyset vs. NetProtect | -1.65471 | 0.23796 | -6.954 | < 0.001 |
| Permanet 2.0 vs. NetProtect | 0.87898 | 0.27272 | 3.223 | 0.00127 |
| Permanet 3.0 vs. NetProtect | 3.20716 | 0.60518 | 5.3 | < 0.001 |
| Permanet 2.0 vs. Olyset | 2.5337 | 0.20789 | 12.188 | < 0.001 |
| Permanet 3.0 vs. Olyset | 4.86188 | 0.57886 | 8.399 | < 0.001 |
| Permanet 3.0 vs. Permanet 2.0 | 2.32818 | 0.59399 | 3.92 | < 0.001 |

Table 2. Pair-wise comparisons, obtained by TukeyHSD test, between the mortality rates of *A. funestus* from Mocuba district exposed to different types of bed nets. P-values adjusted using Westfall procedure implemented with multcomp v. 1.3-7 package.

| **Comparisons** | **Estimate** | **Std. Error** | **z value** | ***P values*** |
| --- | --- | --- | --- | --- |
| Olyset - NetProtect | -1.0239 | 0.1551 | -6.601 | <0.001 |
| Permanet 2.0 vs. NetProtect | 0.973 | 0.1761 | 5.526 | <0.001 |
| Permanet 3.0 vs. NetProtect | 1.6763 | 0.1763 | 9.508 | <0.001 |
| Permanet 2.0 vs. Olyset | 1.9968 | 0.1762 | 11.33 | <0.001 |
| Permanet 3.0 vs. Olyset | 2.7002 | 0.1765 | 15.301 | <0.001 |
| Permanet 3.0 vs. Permanet 2.0 | 0.7033 | 0.1952 | 3.604 | <0.001 |

Table 3. Pair-wise comparisons, obtained by TukeyHSD test, between the mortality rates of *A. funestus* from Balama district exposed to different types of bed nets. P-values adjusted using Westfall procedure implemented with multcomp v. 1.3-7 package.

| **Pair-wise comparisons** | **Estimate** | **Std. Error** | **z value** | ***P values*** |
| --- | --- | --- | --- | --- |
| Olyset vs. NetProtect | -0.7053 | 0.4644 | -1.519 | **0.12886** |
| Permanet 2.0 vs. NetProtect | 1.3468 | 0.4597 | 2.93 | 0.00339 |
| Permanet 3.0 vs. NetProtect | 2.9547 | 0.4908 | 6.02 | < 0.001 |
| Permanet 2.0 vs. Olyset | 2.0521 | 0.4648 | 4.415 | < 0.001 |
| Permanet 3.0 vs. Olyset | 3.66 | 0.4961 | 7.378 | < 0.001 |
| Permanet 3.0 vs. Permanet 2.0 | 1.6079 | 0.4896 | 3.284 | 0.00204 |
