# Supplementary material for: Bio-efficacy of new long-lasting insecticide-treated bed nets against Anopheles funestus and Anopheles gambiae from central and northern Mozambique
Source: Malar J. 2015 Sep 17;14:352. doi: 10.1186/s12936-015-0885-y (PMC4574012; doi:10.1186/s12936-015-0885-y)
Supplement: Supplementary file 2 — Additional file 2: Results of pair-wise comparisons, obtained by TukeyHSD, of mortality rates of mosquitoes exposed to different sides of Permanet 3.0. [file 12936_2015_885_MOESM2_ESM.docx]

Table 1. Results of pair-wise comparisons, obtained by TukeyHSD test, between the mortality rates of *A. gambiae* from Milange district exposed to different sides of new Permanet 3.0. P-values adjusted using Westfall procedure implemented with multcomp v. 1.3-7 package.

| **Comparisons** | **Estimate** | **Std. Error** | **z value** | ***P values*** |
| --- | --- | --- | --- | --- |
| Upper side vs. Lower side | 0.6667 | 1.3877 | 0.480 | 0.633 |
| Roof vs. Lower side | 1.6667 | 1.6204 | 1.029 | 0.561 |
| Roof vs. Upper side | 1.0000 | 1.6736 | 0.598 | 0.561 |

Table 2. Results of pair-wise comparisons, obtained by TukeyHSD test, between the mortality rates of *A. funestus* from Mocuba district exposed to different sides of new Permanet 3.0. P-values adjusted using Westfall procedure implemented with multcomp v. 1.3-7 package.

| **Comparisons** | **Estimate** | **Std. Error** | **z value** | ***P values*** |
| --- | --- | --- | --- | --- |
| Upper side vs. Lower side | 1.250 | 2.704 | 0.462 | 0.64473 |
| Roof vs. Lower side | 11.875 | 3.312 | 3.586 | **0.0013** |
| Roof vs. Upper side | 10.625 | 3.312 | 3.208 | **0.0017** |

Table 3. Results of pair-wise comparisons, obtained by TukeyHSD test, between the mortality rates of *A. funestus* from Balama district exposed to different sides of new Permanet 3.0. P-values adjusted using Westfall procedure implemented with multcomp v. 1.3-7 package.

| **Comparisons** | **Estimate** | **Std. Error** | **z value** | ***P-values*** |
| --- | --- | --- | --- | --- |
| Upper side vs. Lower side | 0.8333 | 4.4656 | 0.187 | 0.852 |
| Roof vs. Lower side | 18.5417 | 5.4692 | 3.390 | **0.003** |
| Roof vs. Upper side | 17.7083 | 5.4692 | 3.238 | **0.003** |
