# Supplementary material for: Bio-efficacy of new long-lasting insecticide-treated bed nets against Anopheles funestus and Anopheles gambiae from central and northern Mozambique
Source: Malar J. 2015 Sep 17;14:352. doi: 10.1186/s12936-015-0885-y (PMC4574012; doi:10.1186/s12936-015-0885-y)
Supplement: Supplementary file 3 — Additional file 3: Ratio between the mortality rate of insectary-susceptible Anopheles arabiensis (Durban strain) and the mortality rate of wild-caught Anopheles funestus (from Balama, Mocuba district) and Anopheles gambiae s.s. from Milange district. The figure shows that the LLINs can still remarkably killing higher number (mortality rate > 90 %) of susceptible mosquitoes. [file 12936_2015_885_MOESM3_ESM.docx]

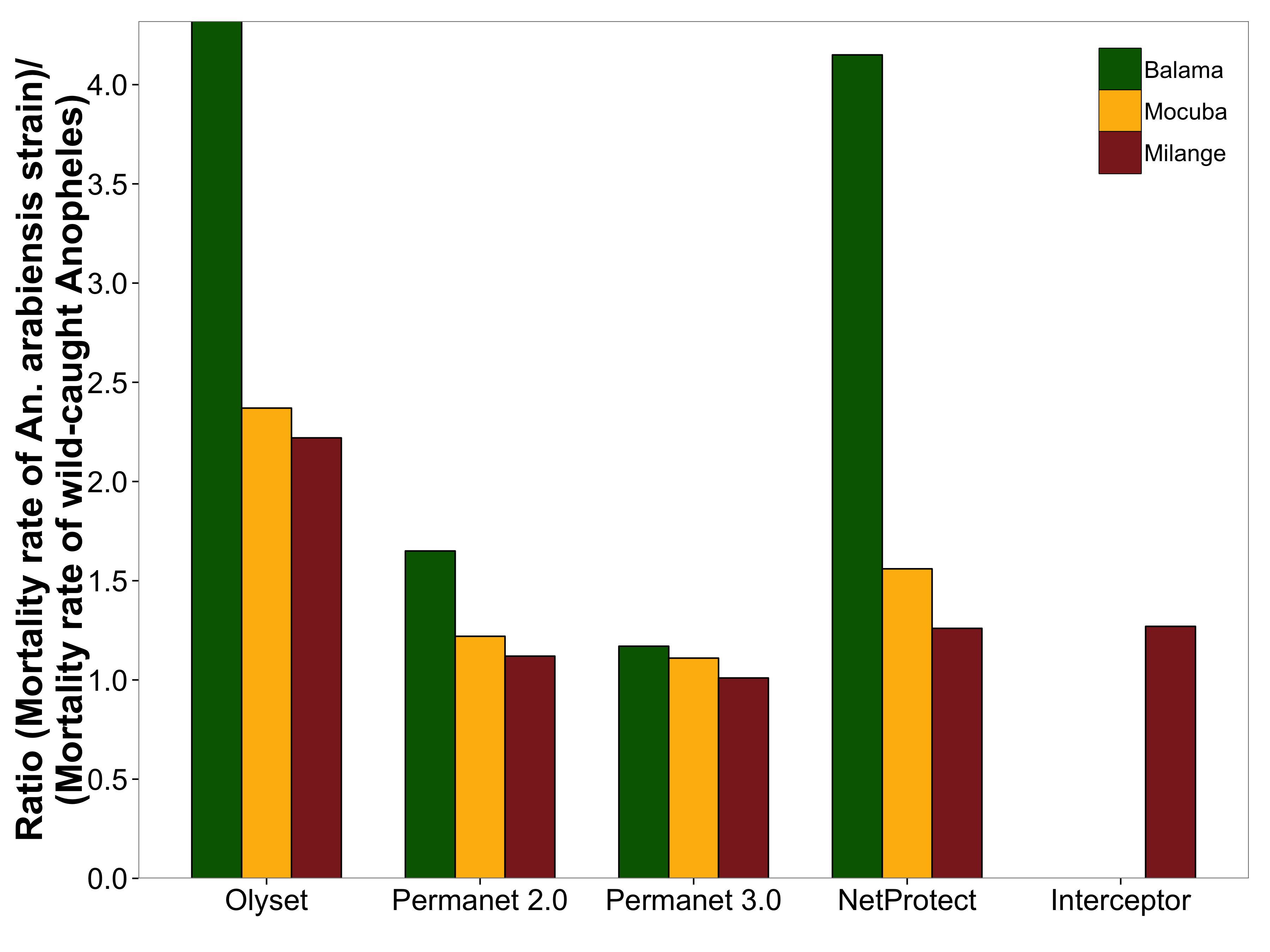


Figure 1. The ratio between mortality rate of susceptible *An. arabiensis* and wild-caught *An. funestus s.s* (Balama and Mocuba) and *An. gambiae s.s*. The figure shows that LLINs, such as Olyset and NetProtect that performed not very well against the field-caught vectors, due to possibility the existence of insecticide resistance strains, can still effectively kill significant number of susceptible mosquitoes.
